# ERL‐ProLiGraph: Enhanced representation learning on protein‐ligand graph structured data for binding affinity prediction

**DOI:** 10.1002/minf.202400044

**Published:** 2024-10-15

**Authors:** Gloria Geine Paendong, Soualihou Ngnamsie Njimbouom, Candra Zonyfar, Jeong‐Dong Kim

**Affiliations:** ^1^ Department of Computer Science and Electronics Engineering Sun Moon University Chungcheongnam-do Korea; ^2^ Department of Computer Science and Engineering Sun Moon University Chungcheongnam-do Korea; ^3^ Genome-Based Bio IT Convergence Institute Sun Moon University Chungcheongnam-do Korea

**Keywords:** binding affinity, bioinformatics, drug discovery, protein ligand interaction

## Abstract

Predicting Protein‐Ligand Binding Affinity (PLBA) is pivotal in drug development, as accurate estimations of PLBA expedite the identification of promising drug candidates for specific targets, thereby accelerating the drug discovery process. Despite substantial advancements in PLBA prediction, developing an efficient and more accurate method remains non‐trivial. Unlike previous computer‐aid PLBA studies which primarily using ligand SMILES and protein sequences represented as strings, this research introduces a Deep Learning‐based method, the Enhanced Representation Learning on Protein‐Ligand Graph Structured data for Binding Affinity Prediction (ERL‐ProLiGraph). The unique aspect of this method is the use of graph representations for both proteins and ligands, intending to learn structural information continued from both to enhance the accuracy of PLBA predictions. In these graphs, nodes represent atomic structures, while edges depict chemical bonds and spatial relationship. The proposed model, leveraging deep‐learning algorithms, effectively learns to correlate these graphical representations with binding affinities. This graph‐based representations approach enhances the model‘s ability to capture the complex molecular interactions critical in PLBA. This work represents a promising advancement in computational techniques for protein‐ligand binding prediction, offering a potential path toward more efficient and accurate predictions in drug development. Comparative analysis indicates that the proposed ERL‐ProLiGraph outperforms previous models, showcasing notable efficacy and providing a more suitable approach for accurate PLBA predictions.

## Graphical abstract

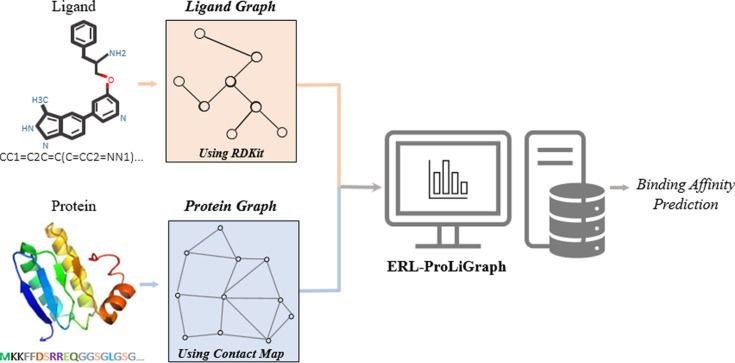

## 1 INTRODUCTION

Proteins, essential components of cellular machinery, interact with molecules known as ligands, a process pivotal to numerous biological functions. The strength of these interactions, quantified as binding affinity, is critical for elucidating molecular behaviors and advancing drug development. Traditionally, protein‐ligand interaction (PLI) and binding affinity prediction have relied on computational methods like molecular docking, free energy calculations, and molecular dynamics simulations. While effective, these approaches are time‐consuming and face challenges in scalability when dealing with large datasets. This context has motivated a shift towards artificial intelligence (AI)‐based models, which promise more efficient and scalable predictions of these interactions.

The advancement of AI, particularly Machine Learning (ML) and Deep Learning (DL), has shown significant potential in bioinformatics [1, 2], revolutionizing conventional analysis methodologies. In the realm of protein‐ligand binding affinity (PLBA) prediction, AI‐based methods excel by automatically extracting features from raw molecular data and learning relevant patterns, leading to faster and more accurate predictions. Central to PLBA prediction is the transformation of complex biological data into formats suitable for AI‐based models. Consequently, a variety of methods emerged for PLBA prediction, aiming to enhance prediction performance using various molecule representation approaches. For instance, a study [3] proposed a spatial extension to the extended connectivity interaction features to improve binding affinity prediction. In addition, structure‐based and ligand‐based features have been shown to contribute to improved PLBA prediction. For example, Boyles et al. demonstrated that ligand‐based features can complement structure‐based scoring functions when trained on docked poses [4]. Profiles of intermolecular contacts have also been utilized for PLBA prediction. Wang et al. [5] developed a prediction method based on profiles of intermolecular contacts. The availability of crystallographic data representation using ML methods has facilitated the development of empirical scoring functions for predicting binding affinity in CDK2‐ligand complexes, which are potential targets for cancer drug discovery [6]. Similarly, Dowker complex‐based machine learning (DCML) models were developed for PLBA prediction [7]. DLSS Affinity, a deep learning model, utilized the pocket–ligand structural pairs as the local information to predict short‐range direct interactions [8].

In recent years, ML and DL approaches have shown promise in improving the prediction of PLBA. A deep Three‐Dimensional Convolutional Neural Network (3D‐CNN) model was utilized to estimate the binding affinity between ligands and biological targets [9], demonstrating its capability to aid the drug discovery pipeline. Another study proposed a deep 3D‐CNN called BindScope to discriminate between active and inactive compounds. KronRLS [10] utilizes kernel‐based regression algorithms to predict compound‐protein interactions and binding affinities. SimBoost [11] leverages gradient‐boosted regression trees and calculate drug‐drug, target‐target, and drug‐target similarity features, significantly improving performance. However, it requires substantial interaction data and does not consider molecular structure. CAPLA [12] uses a cross‐attention mechanism to learn from both protein and ligand sequence‐level information. A DL‐based model called Deep Drug Target Affinity (DTA) [13] uses CNNs to generate representations directly from sequence information of drugs and targets to predict their binding affinities. However, the performance of such models is contingent upon the quality and size of input data. Akin to that, WideDTA [14], a similar model, utilizes CNNs for sequence‐based feature generation but may struggle with limited interaction data and fail to capture complex interactions. Advanced DL frameworks like GANsDTA [15] employ a Generative Adversarial Network for molecular feature learning, offering high predictive efficiency, although computationally intensive and challenging to train. The AttentionDTA [16] model integrates an attention mechanism with CNN to detect relevant patterns and better understand structural aspects of drugs and targets, thereby offering good interpretability. GraphDTA [17] represents the proteins as 1D sequences and the compounds as molecular graphs. GraphDTA utilized Graph Convolution Network (GCN) to learn topological and property features of the drug molecule, demonstrating high performance in DTI predictions. The results of GraphDTA were superior to those of the standard 1D data representation methods, indicating a significant contribution from structural information. However, the paper acknowledges a limitation in their model′s performance. Notably, predicting target affinities is more challenging for certain ligands than others. A small number of ligands contribute disproportionately to the total prediction error, though these ligands are not outliers in the model‘s latent space.

Despite these advancements, the performance of specific models heavily depends on the input features used for training, with frequent uncertainties about their general applicability across various protein‐ligand pairings and molecular structures. This study proposes a graph‐based model called ERL‐ProLiGraph (Enhanced Representation Learning on Protein‐Ligand Graph Structured Data for Binding Affinity Prediction) to address these challenges. ERL‐ProLiGraph aims to improve PLBA prediction performance by representing protein and ligand as molecular graphs, aiding the model to infer the connections between atoms and their bonds directly. The graph representations of molecules offer several advantages in this paper. Firstly, they can capture chemical and structural information of molecules, which is necessary to understand these structures. Secondly, they allow for reconstructing atomic networks that preserve the intricate dynamics and interactions between atoms that might not be feasible with other molecular representations. Furthermore, the paper aims to explore the effectiveness and robustness of other graph‐based methods, such as sample and aggregate (GraphSAGE) and GCN in PLBA prediction.

The paper is structured as follows: Section 2 details the proposed concept and methodology, Section 3 describes the experimental setup, results, and discussion. Finally, Section 4 concludes the paper.

## 2 PREDICTING PROTEIN LIGAND BINDING AFFINITY

**Figure 1:**
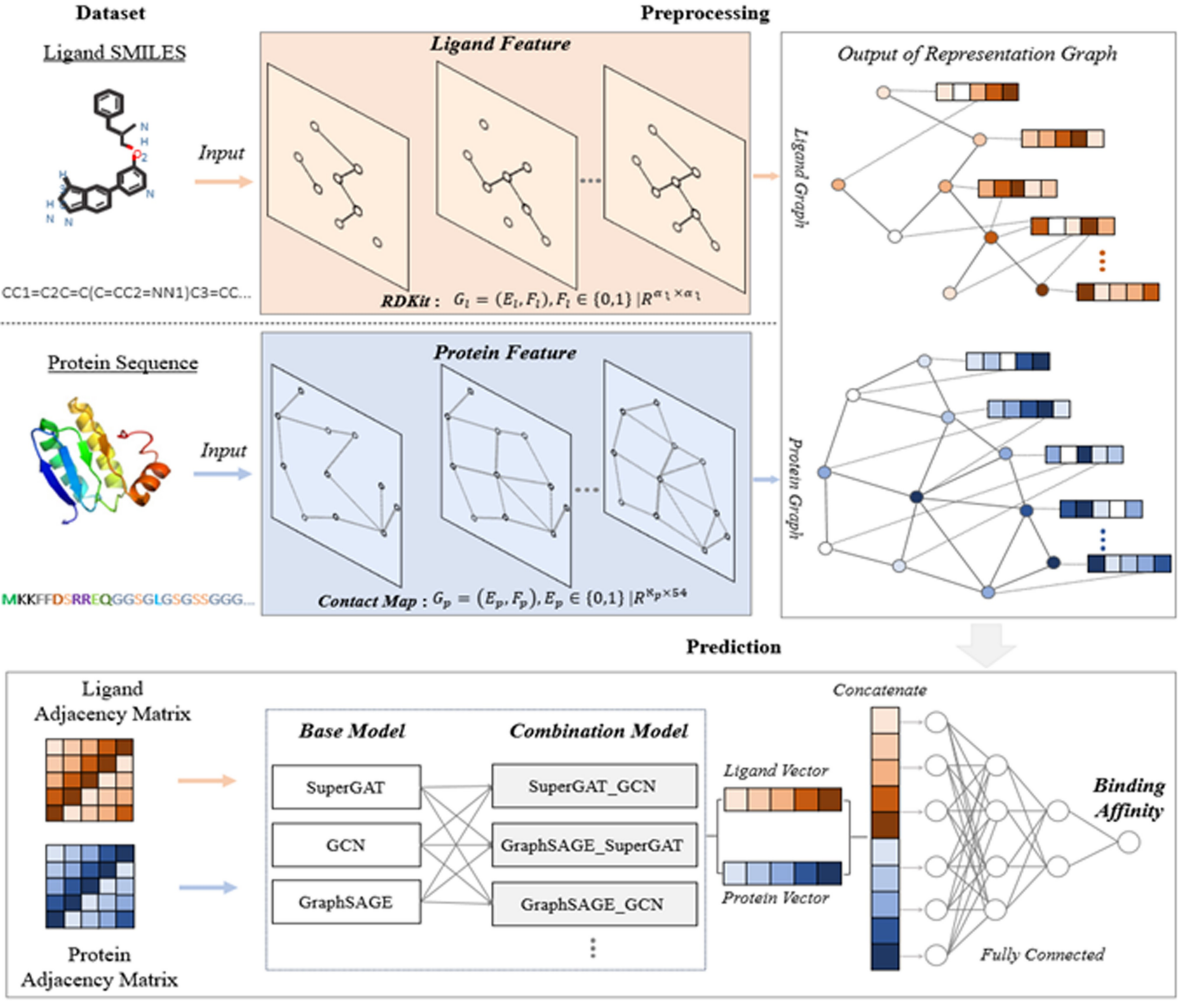
Conceptual model of the proposed ERL‐ProLiGraph model. The model consists of a three‐stage process aimed at predicting protein‐ligand binding affinity. Initially, in the Dataset phase, protein structures and ligand SMILES sequences are collected from the Davis and KIBA datasets for input. During preprocessing, the ligand SMILES are directly converted into molecular graphs using RDKit package, whereas protein graphs are derived from protein contact maps. The prediction phase leverages various combinations of GNN‐based algorithms to extract and learn pivotal features from both protein and ligand graph representations. Finally, these learned features are concatenated and utilized by the classification layer for the precise prediction of binding affinities.

This section details the conceptual framework underlining the development of the proposed ERL‐ProLiGraph model. Concisely, as illustrated in Figure 1, the ERL‐ProLiGraph model consists of three main components: Dataset collection, Preprocessing, and Prediction. The subsequent sections will elaborate on each component of the ERL‐ProLiGraph complex.

### 2.1 Dataset collection

In this study, the training, performance evaluation, and generalization ability of our model in predicting binding affinities were assessed using two prominent datasets: the Davis [18] and KIBA [19] (Kinase Inhibitor Binding Affinity) datasets. The Davis dataset is a well‐established and widely utilized resource in protein‐ligand interaction prediction. It features various protein‐ligand binding affinities sourced from various scientific publications and experimental databases. This extensive collection of experimentally measured binding strengths between various proteins and ligands makes the Davis dataset invaluable for developing and validating models focusing on binding affinity prediction.

Additionally, the KIBA dataset was employed in this research. Tailored for kinase‐inhibitor binding affinity prediction, a key area in drug discovery, the KIBA dataset provides experimentally validated binding affinities, delivering in‐depth insights into kinase‐inhibitor interactions. The emphasis on kinase‐inhibitor interactions in the KIBA dataset is especially pertinent due to the crucial role of kinases in numerous biological pathways and their status as prime drug targets.

Data preparation was carried out to maintain the integrity and consistency of data from both datasets. Following a similar approach to previous studies [13, 18, 20], we removed duplicate drug‐protein pairs from the Davis dataset to avoid label confusion, as the presence of several proteins with identical amino acid sequences could lead to unnecessary repetition in the training data. By eliminating these duplicates, we ensure that our model is trained with cleaner and more representative data, thereby improving accuracy. Such preparation was essential to train and assess the model′s ability to effectively predict binding affinities across different target classes and chemical structures.

### 2.2 Data preprocessing

The following section describes the preprocessing step performed on the input data. The critical step involves converting the data into a format that aligns with the specific requirements of our model‘s input format.

#### 2.2.1 Ligand representation

In this paper, the ligands’ input data were initially in the canonical SMILES format, a format that encodes a molecule‘s structure as a string of characters, with each character or group representing an atom or bond within the molecule. Rather than using the ligand in their original SMILES representation, we leverage the RDKit [21] library to transform the ligands’ SMILES into more complex graph structures. RDKit is a cheminformatics tool widely adopted for molecular representation and manipulation tasks. It offers a rich set of functionalities, including molecular transformations, 2D and 3D coordinate generation, molecular descriptors, and fingerprints, among others, making it the appropriate choice for ligand representation. Its computational efficiency, scalability, and ability to handle large molecular datasets justify its selection for ligand representation in this study [22]. The transformation begins with parsing each SMILES string to construct a corresponding molecule object. This molecule object serves as the basis for further conversion. RDKit is employed to generate a 78‐dimensional feature vector for each atom in the molecule. This vector comprehensively captures various aromatic properties, including atomic number, atom degree, the total and implicit number of hydrogens attached to the atom, and the atom′s aromatic status. These features are detailed in the Table 1. The comprehensive atom and bond information extracted from the molecule object is pivotal for constructing the ligand′s graph representation. In this graph, denoted by $G_{l}=\left(E_{l},F_{l}\right)$ , atoms are represented as nodes, while the bonds linking them are depicted as edges. The adjacency matrix $F_{l}\in \left\{0,1\right\}|R^{\alpha_{l}\times \alpha_{l}}$ represents the chemical bonds between the $\alpha_{l}$ atoms that make up the ligand molecule. Including of self‐loops within the graph structure significantly improves the representation of the ligand molecule′s features 23. This process of constructing the ligand graph from the SMILES sequence is an integral part of the data preprocessing component, as illustrated in Figure 1.

(1) $${G_{l}=\left(E_{l},F_{l}\right),\;F}_{l}\in \left\{0,1\right\}|R^{\alpha_{l}\times \alpha_{l}}$$

**Table 1:** Node features (atom).

**Feature**	**Dimension**
One‐hot encoding of the atom element	44
One‐hot encoding of the degree of the atom in the molecule, which is the number of directly bonded neighbours (atoms)	11
One‐hot encoding of the total number of H bound to the atom	11
One‐hot encoding of the number of implicit H bound to the atom	11
Whether the atom is aromatic	**1**
All	78

#### 2.2.2 Protein representation

During protein representation, Pconsc4 was employed to generate contact maps—which served as a basis for representing proteins as graph data—due to its numerous advantages. Pconsc4 offers accurate and biologically relevant protein contact maps and incorporates evolutionary information to capture essential interactions between protein residues. Its ease of use and efficiency stand out compared to alternative methods such as TripletRes, GREMLIN, CCMpred, SPINE−X, etc. Pconsc4 emerged as the best choice for efficient computational resource usage due to its rapid execution and minimal dependencies, making it the optimal option for our proposed model pipeline [24]. The generated contact maps are 2D encodings of proteins, represented as an adjacency matrix$\left(F_{p}\right).$ This matrix encompasses connectivity information between pairs of amino acids in the protein sequence. For a given protein with ${(ℵ}_{p)}$ residues, the contact map is an output of protein structure prediction, containing distance and connectivity information of the residue pairs. The adjacency matrix is formulated based on the Euclidean distance (${Ed}_{ij})$ between the alpha carbons of the two residues. If this distance is less than or equal to a predefined threshold distance ($d_{threshold})$ , the residues are considered to be in contact, and the corresponding matrix entry is set to 1; otherwise, its value is set to 0. This relationship is represented as follows in Equation (2):

(2) $$F_{p}^{ij}=\left\{\begin{array}{c} 0,\;\;{Ed}_{ij}>d_{threshold}\;∥\;i=j\\ 1,\;\;{Ed}_{ij}\leq d_{threshold} \end{array}\right.$$

Furthermore, the adjacency matrix is combined with an additional 54‐dimensional feature vector, $E_{p}$ $\in \left\{0,1\right\}|R^{{ℵ}_{p}\times 54}$ , which contains complementary node features of the ${ℵ}_{p}$ protein residues to enhance spatial protein representation. The detailed of the node features are summarized in the Table 2. As depicted in Equation (3), the protein graph representation is created from the $E_{p}$ vector and the adjacency matrix ${\;F}_{p}$ .

(3) $${G_{p}\left(E_{p},F_{p}\right),\;E}_{p}\in \left\{0,1\right\}|R^{{ℵ}_{p}\times 54}$$

**Table 2:** Node Features (Residue).

Feature	Dimension
One‐hot encoding of the residue symbol	21
Position‐specific scoring matrix (PSSM)	21
Whether the residue is aliphatic	1
Whether the residue is aromatic	1
Whether the residue is polar neutral	1
Whether the residue is acidic charged	1
Whether the residue is basic charged	1
Residue weight	1
The negative of the logarithm of the dissociation constant for the −COOH group	1
The negative of the logarithm of the dissociation constant for the −NH3 group	1
The negative of the logarithm of the dissociation constant for any other group in the molecule	1
The pH at the isoelectric point	**1**
Hydrophobicity of residue (pH=2)	1
Hydrophobicity of residue (pH=7)	**1**
All	**54**

### 2.3 Prediction methods

With the protein and ligand represented as graphs, our objective is to predict the binding affinity between protein and ligand molecules. To achieve this, DL models were constructed and integrated a range of baseline graph‐based neural networks, including GCN, SuperGAT, and GraphSAGE. Our proposed ERL‐ProLiGraph leverages the strengths of each of the baseline models. Each of these base models, along with their combined models, are described below in section 2.3.1 and 2.3.2. The ERL‐ProLiGraph′s prediction module is presented with pseudocode in the Algorithm.

#### 2.3.1 Base model

##### a GCN

GCN [25] is a specialized type of Graph Neural Network (GNN) that has shown efficacy in predicting binding affinity in protein‐ligand interactions. In molecules′ graph structures, GCNs represent atoms or residues as nodes and the pairwise interactions between them as edges. This design allows GCNs to aggregate information from neighboring nodes, effectively capturing these complex biological systems′ spatial and structural relationships. GCNs have demonstrated an ability to learn from graph structures, offering promises for improved accuracy in predicting binding affinity compared to traditional ML models. The operation of GCN layers is encapsulated in the following Equation 4:

(4) $$H^{\left(l+1\right)}=\;\sigma \;\left(\;{\tilde{D}}^{-\frac{1}{2}}\;\tilde{F}\;{\tilde{D}}^{-\frac{1}{2}}H^{\left(l\right)}W^{\left(l\right)}\right)$$

Where $\tilde{F}$ denotes the adjacency matrix, and $\tilde{D}$ is the degree matrix. $H^{\left(l\right)}$ is the feature representation at layer l , and $W^{\left(l\right)}$ is a layer‐specific trainable weight matrix. The function σ refers to the activation function, typically ReLU, which introduces non‐linearity into the model, allowing the model to capture more complex patterns in the data.

##### b SuperGAT

The Self‐supervised graph attention network (SuperGAT) [26] significantly advanced graph structure learning. It extends the capabilities of traditional Graph Attention Networks by incorporating edge self‐supervision. SuperGAT calculates attention coefficients for each input node i about its neighboring nodes. Equations (5) and (6) represent some abstracted layer working mechanisms of the superGAT model.

(5) $$X_{i}=\;{a_{i,i}\theta X}_{i}+\sum_{j\in ℵ\left(i\right)}{a_{i,j}\theta X}_{j}$$

(6) $$\sigma \left(\sum_{j\in ℵ\left(i\right)}{a_{i,j}WX}_{j}\right)$$

Where 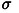 denotes an activation function ReLU and represents the standardized attention coefficients.

##### c GraphSAGE

The GraphSAGE [27], a notable advancement over the original GCN, is designed to learn node representations effectively within large graphs. A key feature of GraphSAGE is its ability to utilize partial graph structure during training while generalizing appropriately to unseen nodes. The operational principle of GraphSAGE involves two main steps: first, it performs neighborhood sampling on the input graph, which helps to control the expansion of receptive fields; second, it learns an aggregation function across multiple search depths. The mathematical representation of the GraphSAGE model is denoted in Equation 7:

(7) $$X_{i}=\;{W_{1}X}_{i}+W_{2•{mean}_{j\in ℵ\left(i\right)}}X_{j}$$

#### 2.3.2 Combination Model

**Figure 2:**
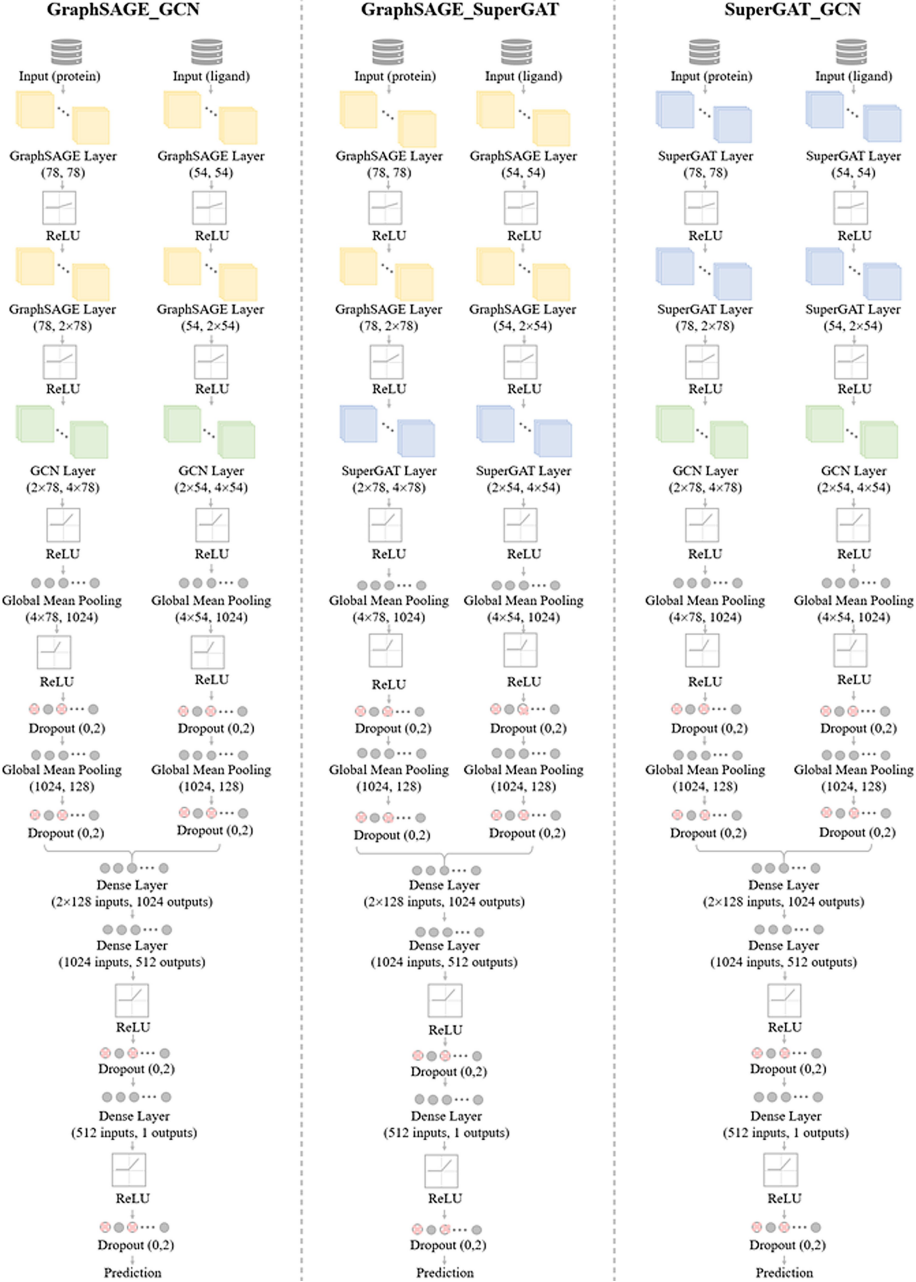
Architecture of the combination models.

The prediction module of the proposed model is a structure composed of the GCN, SuperGAT, GraphSAGE, and combination model. The GCN model can effectively learn graph based structural information, SuperGAT model can learn the association between node attributes through the attention mechanism, and GraphSAGE learn to aggregate feature information from local graph structures. In order to improve the predictive ability of PLBA, we combine the advantages of these models in a combination model module. In combination models, we combine GraphSAGE and GCN (GraphSAGE_GCN), GraphSAGE and SuperGAT (GraphSAGE_SuperGAT), and SuperGAT and GCN (SuperGAT_GCN). These combination model consists of three major components: protein graph processing, ligand graph processing, and combined layers for joint feature extraction. Detailed combination model as shown in Figure 2.

##### a GraphSAGE_GCN

The study presents a deep learning‐based model, GraphSAGE_GCN, for predicting PLBA. The model is designed to handle protein and ligand structures represented as graphs. As shown in Figure 2, the combination module has two branches: one for processing ligand representation data and the other for processing protein data. The ligand processing module includes three GraphSAGEConv and GCNConv layers, progressively capturing hierarchical features of the ligand graph. Following these layers, global mean pooling is applied to obtain a fixed‐size representation. The output is then passed through two fully connected layers with ReLU activation and dropout for further feature refinement. In another branch, the protein processing module comprises GraphSAGEConv and GCNConv layers, capturing essential features from the protein graph. The ligand and protein branches are concatenated and processed through dense layers. This fusion is followed by two fully connected layers with ReLU activation and dropout to extract joint features.

##### b GraphSAGE_SuperGAT

The GraphSAGE_SuperGAT model incorporates the SuperGAT layer, which assigns attention weights to relevant nodes in the protein and ligand graph to handle node classification problems. The model has two input paths: input protein data and input ligand data. Each path follows the same structure, consisting of a GraphSAGE layer, SuperGAT layer, global mean pooling, fully connected layer, ReLu, and dropout. The details are shown in Figure 2.

##### c SuperGAT_GCN

The SuperGAT_GCN model‘s structural configuration is similar to that of the GraphSAGE_GCN and GraphSAGE_SuperGAT models. To improve the model‘s ability to understand the graph‘s local topological structure, a modification is made in the SuperGAT_GCN model. A GCN layer is added after a SuperGAT layer, as visually illustrated in Figure 2. This modification is implemented to further optimize the model‘s ability to discern intricate relationships within the graph‘s local topology.


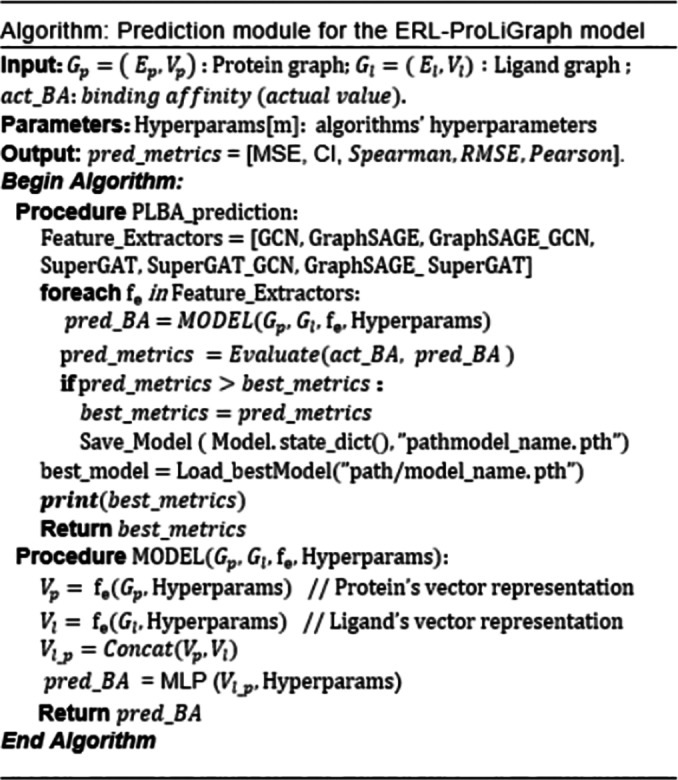


## 3 EXPERIMENTATION

This section provides comprehensive insights into the experimental setup and conditions key to developing our proposed ERL‐ProLiGraph model. It describes the datasets used for training and evaluating the model, detailing their characteristics and ratios. Furthermore, the section elaborates on the results achieved by our ERL‐ProLiGraph model on the prediction of PLBA.

### 3.1 Experimental setup

The experimental setup for developing the proposed ERL‐ProLiGraph model was designed to create a stable and high‐performance computing environment. The hardware configuration includes 64 GB of RAM, an Intel Core i9‐9900k CPU operating at 3.60 Hz, and four NVIDIA GTX 1080 Ti GPUs. The software environment is built on Ubuntu 18.04 64‐bit OS, with Python version 3.11.1. PyTorch Geometric [28] is used for deep learning computations, alongside Cuda version 11.3.1 GPU, and TensorFlow version 2.12.0 for data preprocessing task. The cheminformatics computations are supported by RDKit version 2022.03.5. These components ensure an optimal environment for conducting our computational experiments. The details of these experimental setup are outlined in Table 3.

**Table 3:** Experimental Setup.

Name	Description
RAM	64 GB
CPU	**Intel Core i9‐9900 k (3.60 Hz)**
GPU	**NVIDIA GTX 1080 Ti x 4**
OS	**Ubuntu 18.04 64 bit**
Python Version	**3.11.1**
TensorFlow Version	**2.12.0**
Cuda Version	**11.3.1**
RDKit Version	**2022.03.5**

### 3.2 Hyperparameters setting

Given the significant computational time required for model training, The different hyperparameters were carefully selected based on prior practical experience. The batch size was set to 512 to balance computational efficiency and learning granularity. An Adam optimizer with a learning rate of 0.001 was employed, and the training was conducted over 2000 epochs to guarantee effective learning. Additionally, a dropout rate of 0.2 was implemented to mitigate the risk of overfitting. Regarding the architecture of both the baseline and combination models, we adopted the following configurations: SuperGAT consisted of three layers, which are crucial for capturing complex relationships in graph‐structured data; GraphSAGE, also comprising three layers, GraphSAGE is designed for effective processing of graph data. GraphSAGE_ SuperGAT and SuperGAT_GCN: These combinational components were configured with three layers, optimizing their ability to process and analyze graph data. GraphSAGE_GCN: This combination, with its three layers, enhances the model‘s capacity for in‐depth analysis and pattern recognition. For all the baseline and combination models, two fully connected layers were integrated to perform prediction. These hyperparameters are summarized in Table 4.

**Table 4:** Hyperparameters Setting.

Hyperparameter	Setting
**Epoch**	2000
**Batch size**	512
**Learning rate**	0.001
**Dropout**	**0.2**
**Pooling**	**Global Mean Pooling**
**Optimizer**	**Adam**
**GCN layers**	3
**SuperGAT layers**	3
**GraphSAGE**	3
**GraphSAGE_SuperGAT layers**	3
**SuperGAT_GCN layers**	3
**GraphSAGE_GCN layers**	3
**Fully Connected layers**	2

### 3.3 Datasets

This study utilized two widely recognized datasets, the Davis [18] and KIBA [19] datasets for predicting protein‐ligand interactions.

The Davis dataset provides detailed structural information and interaction analysis encompassing 442 proteins and 68 ligands, recording 30056 interactions. Of these, 2457 are classified as active and 27599 as inactive. Interaction affinity in this dataset is quantified using dissociation constant ($K_{d}$ ) values, which span from 5.0 to 10.8. For computational‐based model prediction, these ($K_{d}$ ) values are converted into a logarithmic scale ($pK_{d}$ ), as per Equation 8:

(8) $$pK_{d}=-\;{log}_{10}\;\left(\frac{K_{d}}{10^{9}}\right)$$

On the other hand, the KIBA dataset provides a collection of kinase inhibitor bioactivities including $K_{i}$ , $K_{d}$ and IC50, which are harmonized into KIBA scores ranging from 0.0 to 17.2. This dataset comprises data on 2111 ligands and 229 proteins, documenting 118254 interactions, with 22729 classified as active and 93426 as inactive.

Each dataset (KIBA and DAVIS) was divided into training and test sets with an 85 : 15 ratio. The training set was utilized for a 5‐fold cross‐validation process; meanwhile, the test set was reserved for evaluating the model‘s performance and assessing its generalizability on unseen data after the completion of model training. During the 5‐fold cross‐validation, the training set was subdivided into 5 folds, with 4 folds used for training and 1‐fold used for validation. This process was repeated 5 times, each time with a different fold used for validation. This process has helped monitor for any potential overfitting during our experiments. Table 5 summarizes the statistics of both datasets.

**Table 5:** Datasets.

Dataset	Ligands	Proteins	Interactions
Davis	68	442	30056
KIBA	2111	229	118254

### 3.4 Evaluation metrics

The performance of our proposed model was evaluated using commonly applied metrics in regression tasks, including Mean Squared Error (MSE), Concordance Index (CI), Pearson Correlation Coefficient (PCC), Spearman Correlation Coefficient (SCC), and Root Mean Squared Error (RMSE).

MSE quantifies the average of the squared differences between the predicted binding affinities (pi ) and the actual values ($y_{i}$ ). It is computed as shown in Equation (9):

(9) $$MSE=\frac{1}{n}\;\sum_{i=1}^{n}{\left(y_{i}-p_{i}\right)}^{2}\;$$

CI measures the discrepancy between the predicted and actual binding affinities. It is calculated using Equation 10:

(10) $$CI=\;\frac{1}{z}\;\sum_{d_{x\;}>{\;d}_{y}}h\left(b_{x}-\;b_{y}\right)$$

The step function h(•) is further detailed in Equation 11:

(11) $$h\;\left(x\right)=\left\{\begin{array}{c} 1,\;if\;x>0\\ 0.5,\;if\;x=0\\ 0,\;if\;x<0 \end{array}\right.$$

The PCC assesses the linear relationship between predicted and actual binding affinities. A value closer to 1 indicates better prediction performance. It is calculated as shown in Equation 12:

(12) $$Pearson=\frac{cov(p,y)}{\sigma \left(p\right)\sigma \left(y\right)}\;$$

The SCC evaluates the monotonic relationship between predicted and actual binding affinities and is calculated using Equation 13:

(13) $$Spearman=1-\frac{6\;\sum d^{2}}{n(n^{2}-1)}$$

Similar to MSE, the RMSE measures the average squared root differences between predicted and actual values. A lower RMSE indicates higher model accuracy and is computed as per Equation 14:

(14) $$RMSE=\sqrt{\frac{1}{n}\;\sum_{i=1}^{n}{\left(pi-yi\right)}^{2}}$$

These metrics collectively provide a comprehensive evaluation of the model‘s performance in terms of accuracy, reliability, and predictive capability.

### 3.5 Results

The proposed ERL‐ProLiGraph model, developed within the PyTorch framework, emerged from a comprehensive series of experiments involving various feature representation learning techniques, such as SuperGAT, GraphSAGE, GCN, SuperGAT_GCN, GraphSAGE_SuperGAT, and GraphSAGE_GCN. Experiments were performed with carefully selected hyperparameters detailed in subsection 3.2. These graph‐based learning representation methods were applied to the protein and ligand graph data to capture the intricate interactions between these molecules, mainly focusing on learning long‐range and spatial interaction features.

Performance evaluations of the ERL‐ProLiGraph model are presented in Tables 6 and 7. These evaluations include the condition where the individual baseline methods and their combinations were used to learn representations in protein and ligand graph‐based structural information. Each variant of the ERL‐ProLiGraph model was assessed on their MSE, RMSE, PCC, SCC, and CI generated scores using the independent test sets from the Davis and KIBA datasets kept aside to measure the model effectiveness after performing a 5‐fold cross‐validation process on the training and validation sets, ensuring the reliability and consistency of the ERL‐ProLiGraph model.

**Table 6:** Results on Davis Dataset.

Methods	MSE	RMSE	PCC	SCC	CI
SuperGAT	0.236	0.486	0.842	**0.695**	0.887
GraphSAGE	0.233	0.483	0.843	0.691	0.889
GCN	0.263	0.513	0.821	0.685	0.880
SuperGAT_GCN	0.252	0.502	0.830	0.675	0.875
GraphSAGE_SuperGAT	0.216	0.465	0.844	0.689	0.890
GraphSAGE_GCN	**0.208**	**0.456**	**0.850**	0.692	**0.895**

**Table 7:** Results on KIBA Dataset.

Methods	MSE	RMSE	PCC	SCC	CI
SuperGAT	0.149	0.385	0.886	0.875	0.885
GraphSAGE	0.146	0.383	0.886	0.881	0.892
GCN	0.150	0.387	0.883	0.874	0.882
SuperGAT_GCN	0.149	0.386	0.888	0.879	0.887
GraphSAGE_SuperGAT	0.154	0.392	0.883	0.878	0.887
GraphSAGE_GCN	**0.137**	**0.369**	**0.897**	**0.884**	**0.893**

Initial experiments involved learning representations of proteins and ligands using baseline methods such as SuperGAT, GraphSAGE, and GCN. The CI scores for these methods varied, ranging from 0.880 to 0.887 for the Davis dataset and from 0.882 to 0.892 for the KIBA dataset. Notably, the GraphSAGE method demonstrated superior results on both datasets, followed by SuperGAT and GCN.

Motivated by these initial results, we furthered our experiments by combining these baseline methods. The combinational methods, namely superGAT_GCN, GraphSAGE_SuperGAT, and GraphSAGE_GCN, exhibited CI scores ranging from 0.875 to 0.895 on the Davis dataset and from 0.887 to 0.893 on the KIBA dataset. Specifically, for the Davis dataset, the GraphSAGE_GCN method achieved the highest performance, with scores of 0.208 (MSE), 0.456 (RMSE), 0.850 (PCC), 0.692 (SCC), and 0.895 (CI). However, the highest SCC score (0.695) was attained using the SuperGAT method. Similarly, for the KIBA dataset, GraphSAGE_GCN demonstrated the highest performance, with scores of 0.137 (MSE), 0.369 (RMSE), 0.897 (PCC), 0.884 (SCC), and 0.893 (CI). GraphSAGE_SuperGAT and SuperGAT_GCN ranked second and third on the Davis dataset among the combinational representation learning methods. Conversely, on the KIBA dataset, SuperGAT_GCN was ranked second, followed by GraphSAGE_SuperGAT in third.

A comparative analysis between the proposed ERL‐ProLiGraph and six state‐of‐the‐art models was conducted utilizing the Davis and KIBA datasets. The evaluation metrics employed were MSE and CI, with results detailed in Tables 8 and 9.

**Table 8:** Comparison of our approach and previous methods on Davis dataset in term of MSE and CI.

**Methods**	**Protein‐ligand rep**.	**CI**	**MSE**
DeepDTA^11^	1D‐PS	0.790	0.608
	SW‐PS	0.886	0.420
	SW‐1D	0.835	0.419
	1D‐1D	0.878	0.261
KronRLS^8^	SW‐PS	0.871	0.379
SimBoost^9^	SW‐PS	0.872	0.282
WideDTA^12^	1D+PDM‐1D+LMCS	0.886	0.262
GANsDTA ^13^	1D‐1D	0.881	0.276
GraphDTA‐GCN ^15^	1D‐Graph	0.880	0.254
**GraphSAGE_GCN**	**Graph‐Graph**	**0.895**	**0.208**

^a^PS: Pubchem Sim; SW: Smith‐Waterman; PDM: Protein Motifs and Domains; LMCS: Live Max Common Substructure.

**Table 9:** Comparison of our approach and previous methods on KIBA dataset in term of MSE and CI.

Methods	Protein‐ligand rep.	CI	MSE
DeepDTA [11]	1D‐PS	0.718	0.571
	SW‐PS	0.710	0.502
	SW‐1D	0.854	0.204
	1D‐1D	0.863	0.194
KronRLS [8]	SW‐PS	0.782	0.411
SimBoost [9]	SW‐PS	0.836	0.222
WideDTA [12]	1D+PDM‐1D+LMCS	0.875	0.179
GANsDTA [13]	1D‐1D	0.866	0.224
GraphDTA‐GAT [15]	1D‐Graph	0.866	0.179
**GraphSAGE GCN**	**Graph‐Graph**	**0.893**	**0.137**

^a^PS: Pubchem Sim; SW: Smith‐Waterman; PDM: Protein Motifs and Domains; LMCS: Live Max Common Substructure.

For the Davis dataset, as shown in Table 8, the ERL‐ProLiGraph model demonstrated superior performance in both CI and MSE metrics compared to other models reported in the literature. Specifically, Regarding CI, the ERL‐ProLiGraph model marks a significant improvement, outperforming the least effective model, DeepDTA [13], by 10.5 % and surpassing the previously top‐performing model, GraphDTA‐GCN [17], by 1.5 %. In terms of MSE, the ERL‐ProLiGraph model displays the lowest error rate of 0.208. This result represents a reduction of 0.4 and 0.046 when compared to the DeepDTA [13] (which had an error rate of 0.608) and GraphDTA‐GCN [17] (which had an error rate of 0.254), respectively.

Similarly, when evaluated on the KIBA dataset (Table 9), the ERL‐ProLiGraph model outperformed other state‐of‐the‐art models. Achieving the highest CI score of 0.893, which surpasses the DeepDTA [13] and GraphDTA‐GCN [17] by significant margins (17.5 % and 2.7 %), respectively. In terms of MSE, the ERL‐ProLiGraph model maintains its leading position by recording the lowest error rate of 0.137. This rate signifies an improvement of 0.434 compared to DeepDTA [13] and 0.042 compared with GraphDTA‐GCN [17] and WideDTA [14].

**Figure 3:**
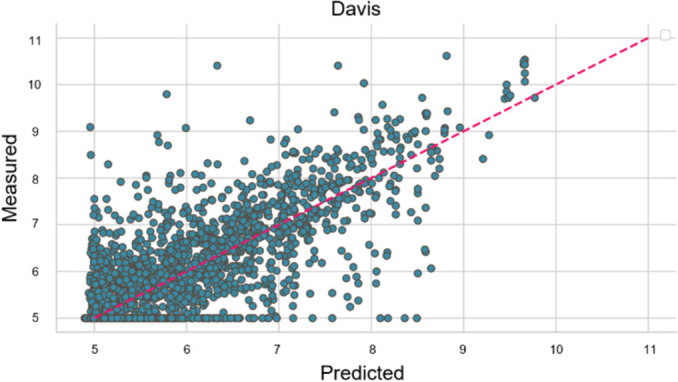
Comparison of predicted and measured binding affinity values for Davis dataset.

**Figure 4:**
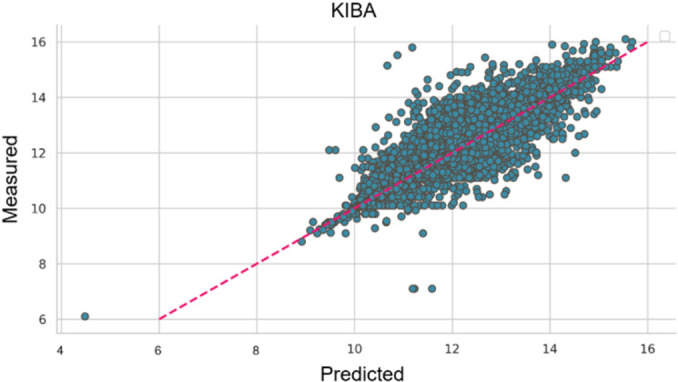
Comparison of predicted and measured binding affinity values for KIBA dataset.

To further demonstrate the prediction accuracy of the proposed ERL‐ProLiGraph model in predicting protein‐ligand binding affinities, we plotted the experimentally verified binding affinities against the binding affinities predicted by our model. These plots, presented for both the Davis and KIBA datasets (Figures 3 and 4, respectively), visually represent the ERL‐ProLiGraph model‘s predictive capabilities. In these plots, the predicted binding affinities (in teal) are closer to the perfect prediction trendline, denoted as 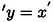 (in red). These alignments suggest the high predictive capacity of the ERL‐ProLiGraph model.

The predictive accuracy of the ERL‐ProLiGraph model is more pronounced when applied to the KIBA dataset than the Davis dataset. This distinction is evidenced by the closer proximity of the ERL‐ProLiGraph’ s predicted values to the ideal prediction trendline in Figure 4 (KIBA) as opposed to Figure 3 (Davis). This observation aligns with the quantitative results we previously reported, where the ERL‐ProLiGraph model achieved higher Pearson and Spearman and lower MSE and RMSE values for the KIBA dataset compared to the Davis dataset.

### 3.6 Discussion

Protein residues exhibit characteristics such as aroma, hydrophobicity, polarity, solubility, and reactivity, which contribute to determining the three‐dimensional structure of proteins and their interactions with other molecules, including ligands in biological systems [29]. These characteristics may manifest in non‐covalent interactions such as hydrophobic forces and hydrogen bonding, and they can influence protein binding. Therefore, this information should be carefully considered when interacting with small molecules. Unfortunately, the PLBA predictions carried out by [12, 15, 30] only consider residue types, treating the sequence as a symbol string, and ignoring important properties. In contrast, ERL‐ProLiGraph, utilizing DL graph‐based, comprehensively extracts spatial structure and attribute information of proteins, along with topological relationships between residues, ensuring that all crucial properties are considered. Based on the reported results, the proposed method performs effectively on both large and small‐scale biomedical data. Evaluation metrics applied to both the KIBA and Davis datasets confirm that ERL‐ProLiGraph exhibits superior performance, outperforming all existing methods in PLBA.

To establish baseline performance, we conducted experiments with a variety of graph‐based algorithms, including GCN [25], SuperGAT [26], and GraphSAGE [27]. These models, each with their unique advantages as discussed in section “2.3.1 Base Model” are not without limitations. For instance, GCN faces challenges in capturing long‐range dependencies within protein structures, a problem highlighted by the findings in Gligorijević’s study [31]. This problem becomes more acute in the context of proteins characterized by complex folds and high contact orders, where GCN′s capability to represent and capture long‐range interactions effectively falls short. SuperGAT′s performance is influenced significantly by the homophily and average degree of the input protein and ligand graphs [32], with its effectiveness being optimal for homogenous graph representations. Such representations, however, offer a simplified structure of protein‐ligand interactions, lacking the depth and complexity needed for accurate modeling. The limitations of SuperGAT become especially apparent when applied to heterogeneous graph representations, which, despite offering a more nuanced representation of protein and ligand molecules— with comprehensive information —pose challenges for this algorithm. GraphSAGE demonstrated superior performances compared to GCN and SuperGAT in our experiments, especially in terms of MSE, RMSE, and CI across the KIBA and Davis datasets. GraphSAGE exhibits weaknesses, particularly its limited generalizability across diverse protein‐ligand complexes and a tendency to struggle with less common protein or ligand structures. Furthermore, its efficiency may decline when processing large and complex graph representations of protein‐ligand interactions, necessitating extended training time, and increased computational resources.

Experimental results show that in terms of ligand‐protein binding affinity with graph data representation, the performance of SuperGAT and GraphSAGE models is superior to GCN. However, when these two models are combined, namely in the form of GraphSAGE_SuperGAT, there is a decrease in performance compared to all other baseline models and model combinations. In contrast, the combination of GraphSAGE and GCN, referred to as GraphSAGE_GCN, shows the best performance as illustrated in Tables 6 and 7.

In this work, protein‐ligand binding affinity prediction is highly dependent on the structural properties of graph data, especially in terms of hierarchical complexity between each node, additionally including node embedding and edge features for enhanced accuracy. These dependencies justify the performance exhibited by the GraphSAGE_GCN, whereby, the GraphSAGE model characterised by its abilities to capture relevant information from the neighbors of each node, is capable to effectively describe the local and hierarchical structures in molecular graphs. And the GCN, known for its ability to aggregate and process features across global graph properties. Therefore, combining the joint abilities of the two methods appears to be highly effective. On the other hand, the SuperGAT, whose prediction mechanism depends on attention‐based feature aggregation, was unable to achieve consistent performance across various datasets. Similarly, the combinational feature extraction GraphSAGE_SuperGAT that used SuperGAT for attention‐based mechanisms was not particularly good at extracting features in dynamically changing graph environments.

While the ERL‐ProLiGraph model has demonstrated remarkable accuracy it has a drawback. Our primary constraint stemmed from the restricted computational resources at our disposal, compelling us to limit the number of layers in our model construction. This decision was driven by the considerable time it took for the model to execute predictions. However, with the advent of a more potent experimental environment equipped with advanced processing units, such as the RTX 3080 Ti, we seized the opportunity to enhance the design of our model‘s construction. This strategic modification is expected to optimize and consequently increase the model‘s performance.

While the ERL‐ProLiGraph model has demonstrated commendable performances, its implementation faced certain limitations. The primary challenge in integrating multiple GNN models was the limited computational resources, necessitating the selection of hyperparameters based on prior experience rather than utilizing established hyperparameter tuning algorithms such as random or grid search due to the complexity of the ERL‐ProLiGraph model. This decision was influenced by the considerable time required for the model to make predictions. Consequently, the potential sensitivity of the proposed model to the chosen hyperparameters could not be evaluated. With more advanced experimental environments equipped with high‐performance processing units like the RTX 3080 Ti, we could improve the model‘s architecture and optimize hyperparameters, potentially enhancing the model‘s performance. Additionally, we did not conduct an interpretability analysis of the proposed model, which is essential for experimental chemistry research. Recognizing this analysis‘s importance in increasing the model‘s trustworthiness, we commit to incorporating interpretability analysis in future work.

## 4 CONCLUSIONS

The research proposes an end‐to‐end DL approach to predict binding affinity between protein and ligand molecules. Conventional PLBA prediction models employ CNNs and RNNs‐based algorithms to extract features from Euclidean data representations, such as 1D sequences, 2D grid images, or 3D grids mapping atom density. However, these representations may not fully capture the spatial relationships and interactions among atoms in protein sequences and ligand molecules. In contrast, the present work utilizes graph‐based representations derived from predicted contact maps, which encode the pairwise residue‐residue contacts and spatial information of the 3D protein structure. These representations, structured as adjacency matrices, offer a non‐Euclidean data representation that effectively retains the spatial information among atoms. The proposed approach utilized graph‐based neural networks, such as GCN, GraphSAGE, SuperGAT, and their synergistic combinations, which are proficient at extracting meaningful features from this type of data representation. During experimentation, we explored baseline and combination models to determine the most efficient feature extraction method. Our findings revealed that the model combining GraphSAGE and GCN achieved the highest predictive performance. The ERL‐ProLiGraph was evaluated on two well‐recognized datasets in PLBA prediction, and performance comparisons indicated that our ERL‐ProLiGraph model surpassed most state‐of‐the‐art PLBA prediction models. This achievement is particularly significant for the pharmaceutical industry, constantly seeking innovative approaches to expedite drug development processes. The demonstrated efficacy of the ERL‐ProLiGraph model suggests its transformative potential in this field, contributing to enhance efficiency and reliability in drug discovery.

## SUPPORTING INFORMATION

Additional supporting information can be found online in the Supporting Information section at the end of this article.

## Conflict of Interests

The authors declare no conflicts of interest.

5

## Supporting information

As a service to our authors and readers, this journal provides supporting information supplied by the authors. Such materials are peer reviewed and may be re‐organized for online delivery, but are not copy‐edited or typeset. Technical support issues arising from supporting information (other than missing files) should be addressed to the authors.

Supporting Information

## Data Availability

The data that support the findings of this study are openly available at https://doi.org/10.1093/bioinformatics/bty593, reference number [13]. The material used in our work is available at https://github.com/glorygeine/ERL‐ProLiGraph/tree/main.
