# Supplementary material for: ERL‐ProLiGraph: Enhanced representation learning on protein‐ligand graph structured data for binding affinity prediction
Source: Mol Inform. 2024 Oct 15;43(12):e202400044. doi: 10.1002/minf.202400044 (PMC11639045; doi:10.1002/minf.202400044)

molecular informatics

Supporting Information

ERL-ProLiGraph: Enhanced representation learning on protein-ligand graph structured data for binding affinity prediction

Gloria Geine Paendong[1] | Soualihou Ngnamsie Njimbouom[1] | Candra Zonyfar[1] | Jeong-Dong Kim[1,2,3]

Graphical TOC / Abstract

Title:

ERL-ProLiGraph: Enhanced Representation Learning on Protein-Ligand Graph Structured Data for Binding Affinity Prediction

Authors:

Gloria Geine Paendong[a], Soualihou Ngnamsie Njimbouom[a], Candra Zonyfar[a] and Jeong-Dong Kim*[a,b,c]

Key Findings:

- An end-to-end graph-based model (ERL-ProLiGraph) to accurately predict protein-ligand binding affinity.

- Capturing intricate molecular interactions from protein and ligand graph-based representations.

- Leveraging GCN, GraphSAGE, SuperGAT and their various combinations to extract pivotal features from protein and ligand graph representations.

Graphical Abstract:

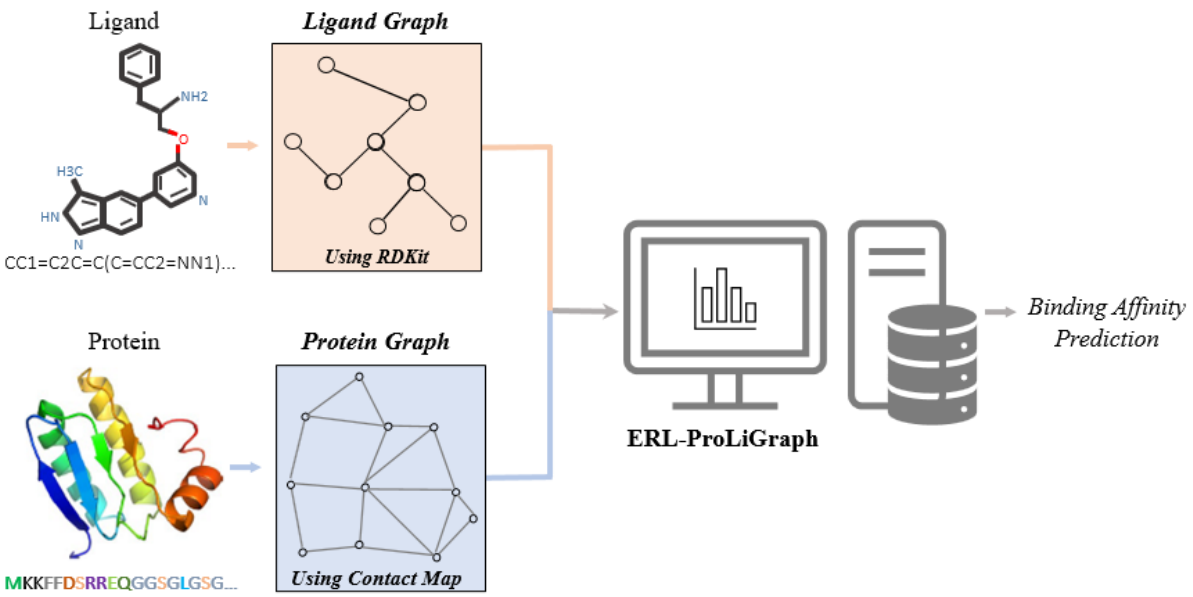

Supplement: Supplementary file 1 — Supporting Information [file MINF-43-e202400044-s001.pdf]
